# Cytokine-Leukotriene Receptor Interactions

**DOI:** 10.1100/tsw.2007.183

**Published:** 2007-09-01

**Authors:** Marek Rola-Pleszczynski, Jana Stankova

**Affiliations:** Immunology Division, Department of Pediatrics, Faculty of Medicine, Université de Sherbrooke, Sherbrooke QC, J1H 5N4, Canada

**Keywords:** leukotriene, lipid mediator, receptor, G-protein coupled receptor, arachidonic acid, cytokine, interleukin, interferon, growth factor, signaling

## Abstract

Biochemical and pharmacological studies have identified the structure of leukotrienes, the pathways that lead to their synthesis, and the signaling events they trigger when they interact with their cognate receptors. A privileged interaction exists between these lipid mediators and another group of molecules essential for inflammation and immune modulation, namely, cytokines. Whereas leukotrienes can trigger the synthesis and release of selected cytokines in distinct cell populations, many cytokines can affect cellular responsiveness to leukotrienes by modulating leukotriene receptor expression. As we progressively begin to unravel these complex interactions, new areas of cell-cell communication and eventual therapeutic interventions will emerge.

Receptors for leukotriene (LT) $B_4$ ($BLT_1$ and $BLT_2$) and for the cysteinyl-LTs ($CysLT_1$ and $CysLT_2$) had been studied pharmacologically for almost 2 decades before they were cloned and structurally characterized. In this paper, we will review how leukotrienes can modulate cytokine production through their interaction with selective receptors and, conversely, how various cytokines can regulate the expression of leukotriene receptors and thus affect cellular responsiveness to leukotrienes.

### MODULATION OF CYTOKINE PRODUCTION BY LEUKOTRIENE RECEPTOR ACTIVATION

#### Leukotriene $B_4$

Soon after the discovery of leukotrienes by Borgeat and Samuelsson[1,2], $LTB_4$ was found to modulate the immune response by inducing suppressor cells and by enhancing natural cytotoxic activity in lymphocytes[3,4,5]. $LTB_4$ was also shown to stimulate the production of interleukin (IL)-2, interferon (IFN)γ, and IL-5 by T cells[6,7,8,9] (Table 1).

$LTB_4$ stimulation of human monocytes was found to activate gene transcription, namely, that of the transcription factors c-fos and c-jun[10]. Moreover, $LTB_4$ could induce the transcriptional activation of the IL-6 gene and enhance IL-6 production in monocytes[11,12]. Macrophages stimulated with platelet-activating factor (PAF) produced increased amounts of IL-6 and TNFα, both of which were dependent on endogenous leukotriene production[13].

**TABLE 1**
**Leukotriene-Induced Modulation of Cytokine Expression**

| Leukotriene | Cytokine | Effect* | Target Cells | Ref. |
|---|---|---|---|---|
| $LTB_4$ | IL-2 | + | T lymphocytes | [7] |
| | IFNγ | + | T lymphocytes | [6,7,8] |
| | IL-5 | + | T lymphocytes | [9] |
| | IL-6 | + | Monocytes, macrophages | [11,12] |
| | TNFα | + | Alveolar macrophages | [13] |
| | MCP-1 | + | Monocytes | [16] |
| | IL-8 | + | PMN | [17] |
| | IL-10 | + | Dendritic cells (mouse) | [19] |
| | IL-2Rα | + | Monocytes | [15] |
| | IL-2Rβ | + | Monocytes, NK cells | [14,15] |
| | LIF | + | BM stromal cells | [18] |
| $LTC_4$, $LTD_4$ | IL-8 | + | Monocytes, dendritic cells, endothelial cells, mast cells | [20,31,32] |
| | MCP-1 | + | Monocytes, THP-1 cells | [21,22] |
| | RANTES | + | Lung mononuclear cells | [23] |
| | MIP-1β | + | Alveolar macrophages, mast cells | [24,30] |
| | IL-1β | + | Vascular smooth muscle cells | [29] |
| | TNFα | + | Alveolar macrophage, mast cells | [24,30] |
| | IL-4 | + | Eosinophils | [33,34] |
| | IL-5 | + | Mast cells | [30] |
| | IL-10 | + | Dendritic cells | [35] |
| | TGFβ | + | Eosinophils, airway epithelial cells | [25,26,28] |

* The plus (+) sign indicates augmentation of production of the corresponding cytokine.

Human natural killer (NK) cells can be activated by IL-2. $LTB_4$ was found to up-regulate the expression of the β chain of the IL-2 receptor, thus rendering the cells more responsive to the stimulatory effects of IL-2[14]. NK cells pre-exposed to $LTB_4$ became more cytotoxic to target cells when treated with IL-2 than unexposed NK cells.

Resting monocytes do not express the α chain of the IL-2 receptor complex. $LTB_4$ was shown to induce the expression of IL-2Rα and to augment the expression of IL-2Rβ in human monocytes[15], thus allowing the cells to express the heterotrimeric, high-affinity IL-2 receptor complex. $LTB_4$-pretreated monocytes were thus rendered more sensitive to IL-2 and consequently produced greater amounts of TNFα in response to lower concentrations of IL-2.

$LTB_4$ was also found to up-regulate the expression of monocyte chemoattractant protein-1 (MCP-1) in monocytes[16] and IL-8 in neutrophils[17].

$LTB_4$ was reported to induce leukemia inhibitory factor (LIF), but not IL-6 production by human bone marrow stromal cells[18]. In murine bone marrow–derived dendritic cells, $LTB_4$ enhanced LPS-stimulated IL-10 release by approximately 40% and inhibited IL-12 p40 release by approximately 20%[19]. Both effects were mediated by the high-affinity $BLT_1$ receptor.

### Cysteinyl-Leukotrienes

Because cysteinyl-leukotrienes (CysLTs) are major protagonists in the pathophysiology of human asthma, and because neutrophils are involved in the more severe form of asthma, we studied the potential for $LTD_4$ to induce synthesis of the chemokine IL-8 through activation of the $CysLT_1$ receptor. We found $LTD_4$ to induce IL-8 gene expression in monocytic THP-1 cells and human dendritic cells with complete abrogation by selective $CysLT_1$ antagonists[20]. Human embryonic kidney (HEK)-293 cells stably transfected with $CysLT_1$ were used to further study the transcriptional regulation of the IL-8 promoter. Stimulation of the cells with graded concentrations of $LTD_4$ resulted in a time- and concentration-dependent induction of IL-8 transcription and protein synthesis. Use of IL-8 promoter mutants with substitutions in their NF-κB, activator protein (AP)-1, and NF-IL-6 binding elements revealed a requirement for NF-κB and AP-1, but not NF-IL-6, in $LTD_4$-induced activation of the IL-8 promoter. Overexpression of dominant-negative IκB inhibited the IL-8 transactivation induced by $LTD_4$. NF-κB DNA binding activity was induced by $LTD_4$, as determined by electrophoretic mobility shift assays, and could be supershifted by antibodies against p50 and p65. Supershift assays after $LTD_4$ stimulation also indicated the formation of a c-Jun/c-Fos complex. Moreover, our results demonstrate that $LTD_4$ up-regulates the expression of c-*fos* and *c-jun* at the mRNA level. Our data showed for the first time that $LTD_4$, via the CysLT1 receptor, could transcriptionally activate IL-8 production, with involvement of the transcription factors p50, p65, Fos, and Jun. These findings provide mechanistic and potentially therapeutic elements for modulation of the inflammatory component of asthma.

In this context, CysLTs have been reported to induce the production of other chemokines, such as monocyte chemoattractant protein (MCP)-1 in human monocytes/macrophages[21,22] and Regulated upon Activation, Normal T-cell Expressed, and Secreted (RANTES) in a murine model of asthma[23]. In a rat alveolar macrophage line, $LTD_4$ enhanced mRNA and protein expression of MIP-1α and TNFα induced by LPS[24].

CysLTs have also been shown to induce or augment the expression of TGFβ in eosinophils and airway epithelial cells[25,26]. We have recently confirmed these findings and extended them to show that $LTD_4$-induced TGFβ can synergize with fibroblast-growth factor-2 to induce bronchial smooth muscle cell proliferation[27,28]. This further suggests a mechanism for the airway remodeling potential of CysLTs. The latter may also be involved in inflammatory phenomena within the vessel wall. Indeed, in vascular smooth muscle cells, $LTD_4$ was found to induce IL-1β production[29], suggesting that it may play a role in the pathogenesis of atherosclerosis.

IL-4–primed mast cells were shown to secrete IL-5, TNFα, and MIP-1β when stimulated with either $LTC_4$ or $LTD_4$[30]. Moreover, the $CysLT_1$ antagonist MK571 significantly attenuated the generation of IL-5 and TNFα by mast cells activated by FcεRI cross-linkage[30], suggesting that endogenous CysLTs also participated in IgE-dependent cytokine production in these cells via the $CysLT_1$ receptor. The same group also indicated that IL-8 secretion was stimulated by $LTC_4$ and $LTD_4$ in IL-4–primed mast cells (although no baseline IL-8 production was shown)[31]. In contrast to the other cytokines, however, IL-8 secretion was reportedly resistant to blockade by MK571, suggesting dependence on the $CysLT_2$ receptor for LT-induced mast cell production of this cytokine. We have also recently shown that $LTC_4$, more than $LTD_4$, could induce IL-8 production in endothelial cells, expressing exclusively $CysLT_2$[32]. Moreover, in cells stably transfected with $CysLT_2$, $LTC_4$ induced IL-8 promoter transactivation, which was dependent on AP-1 and NF-κB transcription factors and which involved PKC δ and ε signaling, respectively.

$LTC_4$ was shown to induce IL-4 production in cord blood eosinophils[33] and to induce IL-4 release from blood eosinophils through an intracrine mechanism[34]. CysLTs were also found to regulate dendritic cell functions in a murine model of asthma and to modulate the production of IL-10 and IL-5 in the lungs[35]. Conversely, the $CysLT_1$ antagonist pranlukast was shown to inhibit IL-5 production in rats[36] and guinea pigs[37] in models of antigen-dependent late asthmatic response. Pranlukast was also shown to attenuate allergen-specific TNFα production and NF-κB translocation in monocytes of asthmatic patients[38].

Of note, some effects of $CysLT_1$ antagonists have been shown to be independent of their receptor antagonist activity, especially at higher concentrations. Thus, they can modulate iNOS function[39], reduce IL-5 production[40], and inhibit NF-κB activation[41].

### MODULATION OF LEUKOTRIENE RECEPTOR EXPRESSION BY CYTOKINES

Since the level of expression of receptors for inflammatory mediators may directly affect the degree of responsiveness of cells to these mediators, we and others have studied the potential for modulation of leukotriene receptor expression by biological response modifiers, including cytokines.

### $BLT_1$

Although TNFα was reported to modulate the affinity state of the $LTB_4$ receptor in human PMN[42], the expression of the high-affinity receptor for $LTB_4$ ($BLT_1$) has been found to be relatively resistant to regulation. When they characterized the promoter region of the $BLT_1$ gene, Kato and colleagues[43] found that it contains cis-elements mainly associated with basal expression, such as SP1, which was found to be a major activator of basal transcription of $BLT_1$. The promoter region of the $BLT_1$ gene includes the open reading frame (ORF) of the $BLT_2$ gene, which encodes a low-affinity receptor for $LTB_4$[44]. $BLT_1$ is predominantly expressed on leukocytes and endothelial cells. Interestingly, the CpG sites of the $BLT_1$ promoter region are highly methylated in $BLT_1$-nonexpressing cells, but not methylated in $BLT_1$-expressing cells. Furthermore, methylation of this region *in vitro* inhibited the promoter activity of the $BLT_1$ gene. Thus, methylation at CpG sites in the promoter region is important for cell-specific transcription of $BLT_1$.

We have shown $BLT_1$ expression in human neutrophils to be up-regulated by the corticosteroid dexamethasone (DEX)[45] (Table 2). DEX up-regulated the steady-state levels of $BLT_1$ mRNA in human neutrophils in a time- and concentration-dependent manner. The effect was dependent on transcriptional activity, whereas $BLT_1$ mRNA stability was not affected. DEX-induced up-regulation of $BLT_1$ expression was prevented by pretreatment with the $LTB_4$ antagonist LY 255283. Moreover, $LTB_4$ itself up-regulated the expression of $BLT_1$ mRNA. $BLT_1$ protein expression on neutrophils exposed to DEX for 24 h was also up-regulated two- to threefold, and DEX-treated as well as $LTB_4$-treated cells showed enhanced responsiveness to $LTB_4$ in terms of intracellular $Ca^{++}$ mobilization and chemotaxis. Whereas DEX and $LTB_4$ alone decreased neutrophil apoptosis by approximately 50%, neutrophils treated with both $LTB_4$ and DEX showed greater than 90% survival at 24 h. Moreover, $BLT_1$ antagonists prevented the increased neutrophil survival induced by DEX as well as by $LTB_4$. Taken together, our results suggest that DEX-induced up-regulation of $BLT_1$ expression in neutrophils may be one mechanism through which glucocorticoids can prolong neutrophil survival, namely, by enhancing cell responses to the antiapoptotic effect of $LTB_4$.

Pettersson and colleagues[46] reported that proinflammatory mediators, such as IFNγ, TNFα, and LPS, down-regulated $BLT_1$ expression in monocytes, whereas the anti-inflammatory cytokine, IL-10, and DEX up-regulated $BLT_1$ expression. The effect of IFNγ appeared to be exerted through a block in transcriptional activity. Receptor down-modulation following IFNγ stimulation resulted in a diminished chemotactic response to $LTB_4$. In our hands, however, $BLT_1$ expression in monocytes, macrophages, or neutrophils was unaffected by any of a number of stimuli, including the bacterial products LPS and formyl-methionyl-leucyl-phenylalanine, or the cytokines IL-1β, TNFα, IL-4, IL-6, IL-10, or GM-CSF (unpublished observations).

**TABLE 2**
**Cytokine-Induced Modulation of Leukotriene Receptor Expression**

| Leukotriene Receptor | Cytokine | Effect* | Target Cell | Ref. |
|---|---|---|---|---|
| $BLT_1$ | IL-1β | + | Endothelial cells | [47] |
| | TNFα | Weak | Endothelial cells | [47] |
| | | –, or 0 | Monocytes | [46], MR-P, unpublished |
| | IL-10 | +, or 0 | Monocytes | [46], MR-P, unpublished |
| | IFNγ | –, or 0 | Monocytes | [46], MR-P, unpublished |
| | SCF | – | Mast cells | [48] |
| $BLT_2$ | TNFα | + | Endothelial cells | [47] |
| | IL-1β | + | Endothelial cells | [47] |
| | SCF | – | Mast cells | [48] |
| $CysLT_1$ | IL-5 | + | Eosinophilic HL-60 cells | [52] |
| | IL-4 | + | Monocytes, macrophages; B and T lymphocytes | [54,55,60] |
| | | 0 | Mast cells | [56] |
| | | 0 | Bronchial smooth muscle cells | [57] |
| | IL-13 | + | Monocytes, macrophages; bronchial smooth muscle cells; fibroblasts; eosinophils | [54,55,57,59] |
| | IFNγ | + | Bronchial smooth muscle cells | [57,58] |
| | | – | Macrophages (inhibits IL-4 effect) | MR-P, unpublished |
| | TGFβ | + | Bronchial smooth muscle cells | [57] |
| $CysLT_2$ | IL-4 | + | Mast cells; T and B lymphocytes; eosinophils | [31,54] |
| | | 0 | Eosinophils | [63] |
| | IFNγ | + | Eosinophils; endothelial cells; monocytes; T and B lymphocytes | [54,63,64] |
| | | 0 | Bronchial smooth muscle cells | [57] |
| | IL-13 | + | Eosinophils | [54] |
| | | 0 | Bronchial smooth muscle cells, eosinophils | [57,63] |
| | TGFβ | 0 | Bronchial smooth muscle cells | [57] |

* The plus (+) sign indicates augmentation of receptor expression in the indicated cells by the corresponding cytokine, whereas the negative (–) or zero (0) sign indicates reduction of expression or no effect, respectively.

On the other hand, endothelial cells, which normally express very little $BLT_1$, were recently shown to express $BLT_1$ in response to LPS, $LTB_4$, and IL-1β[47]. Moreover, stem cell factor was shown to inhibit $BLT_1$ mRNA expression in murine bone marrow-derived mast cells and reduce their migration to $LTB_4$[48].

### $BLT_2$

$BLT_2$, which binds $LTB_4$ with a lower affinity, is expressed ubiquitously, in contrast to $BLT_1$. Since its open reading frame is located in the promoter region of the $BLT_1$ gene, it is suggested that there may be shared transcriptional regulation of these two receptors[44]. Very little information is available on the modulation of $BLT_2$ expression. Treatment of human umbilical vein endothelial cells with LPS leads to a greater than tenfold increase in the levels of $BLT_1$ mRNA without any significant effects on $BLT_2$ mRNA[47]. TNFα increases the expression of $BLT_2$ mRNA approximately six times above basal levels with only a modest increase in $BLT_1$ mRNA. IL-1β causes variable and parallel increases of both $BLT_1$ and $BLT_2$ mRNA. On the other hand, stem cell factor was found to reduce $BLT_2$ (and $BLT_1$) mRNA expression in murine bone marrow–derived mast cells[48].

In conclusion, although $LTB_4$ can modulate the production of a number of cytokines and thus appear to play an important role in inflammation and immune defenses, the expression of its receptors is relatively resistant to modulation by cytokines or other biological modifiers. This allows $BLT_1$-expressing cells to maintain a consistent responsiveness to $LTB_4$ with relative independence from environmental influences.

### $CysLT_1$

$CysLT_1$ expression in healthy individuals appears to be fairly restricted to leukocytes and bronchial smooth muscle cells (BSMC)[49,50]. During inflammation, however, other cells, e.g., bronchial epithelial cells, have also been found to express $CysLT_1$[51].

Modulation of $CysLT_1$ expression was first demonstrated using the cytokine IL-5[52]. We had previously shown that HL-60 cells differentiated toward the eosinophilic lineage (HL-60/eos) developed specific functional $LTD_4$ receptors[53]. Work was then undertaken to study the potential modulation of $CysLT_1$ expression in HL-60/eos by IL-5, an important regulator of eosinophil function. IL-5 could rapidly up-regulate $CysLT_1$ mRNA expression, with consequently enhanced $CysLT_1$ protein expression and function in HL-60/eos. $CysLT_1$ mRNA expression was augmented two- to 15-fold following treatment with IL-5. The effect was seen after 2 h, was maximal by 8 h, and maintained at 24 h. Although $CysLT_1$ mRNA was constitutively expressed in undifferentiated HL-60 cells, its expression was not modulated by IL-5 in the absence of differentiation. Differentiated HL-60/eos cells pretreated with IL-5 for 24 h showed enhanced $CysLT_1$ expression on the cell surface, as assessed by flow cytometry using a polyclonal anti-$CysLT_1$ antibody. They also showed enhanced responsiveness to LTD4, but not to $LTB_4$ or PAF, in terms of $Ca^{++}$ mobilization, and augmented chemotactic response to LTD4. Recently, Early and colleagues[54] reported that IL-13 could also augment $CysLT_1$ expression in eosinophils.

In a subsequent study, we also reported that the Th2 cytokine IL-13 could upregulate $CysLT_1$ mRNA levels, with consequently enhanced $CysLT_1$ protein expression and function in human monocytes and monocyte-derived macrophages[55]. $CysLT_1$ mRNA expression was augmented two- to fivefold following treatment with IL-13 and was due to enhanced transcriptional activity. The effect was observed after 4 h, was maximal by 8 h, and maintained at 24 h. IL-4, but not IFNγ, induced a similar pattern of $CysLT_1$ up-regulation. Interestingly, IFNγ could prevent the upregulation of $CysLT_1$ mRNA induced by IL-4 or IL-13.

Monocytes pretreated with IL-13 or IL-4 for 24 h also showed enhanced $CysLT_1$ protein expression, as assessed by flow cytometry using a polyclonal anti-$CysLT_1$ antibody[55]. They also showed enhanced responsiveness to LTD4, but not to $LTB_4$, in terms of $Ca^{++}$ mobilization, as well as augmented chemotactic activity. These studies suggested a possible mechanism by which IL-5, IL-13, and IL-4 could modulate $CysLT_1$ expression on eosinophils, monocytes, and macrophages, and consequently their responsiveness to $LTD_4$, and thus contribute to the pathogenesis of asthma and allergic diseases. Woszczek and colleagues[22] confirmed our findings with IL-4 and determined the activation of a STAT6 responsive element in the $CysLT_1$ promoter by IL-4. Interestingly, treating cord blood–derived mast cells with IL-4 did not alter $CysLT_1$ receptor mRNA or cell surface protein expression[56].

Airway remodeling is a feature of chronic asthma and involves a number of structural changes, including BSMC hyperplasia and hypertrophy. CysLTs have been suggested to play a role in airway remodeling in addition to their numerous other physiopathological effects. In a subsequent study[57], we aimed at characterizing the potential modulation by cytokines of $CysLT_1$ receptor expression in BSMC and the eventual functional relevance of this modulation. When human BSMC were exposed to TGFβ, IL-13, or IFNγ, their expression of $CysLT_1$ receptor was significantly augmented in a time- and concentration-dependent manner. The positive effect of IFNγ on BSMC proliferation was also reported by Amrani and colleagues[58]. Interestingly, IL-4 had no significant effect on $CysLT_1$ receptor expression in BSMC. Moreover, IL-13 and IFNγ, but not TGFβ, were able to increase $CysLT_1$ mRNA levels. Finally, when BSMC were pretreated with TGFβ or IL-13, but not IFNγ, their responsiveness to $LTD_4$ was markedly enhanced in terms of BSMC proliferation. Whereas TGFβ, IL-13, or $LTD_4$ alone had little effect on BSMC proliferation, pre-exposure of the cells to TGFβ or IL-13 for 24 h resulted in a significant increase in proliferation in response to $LTD_4$. The enhanced proliferation was totally prevented by pretreating the cytokine-primed BSMC with the selective $CysLT_1$ receptor antagonist, montelukast. Taken together, these findings indicated a synergy between certain cytokines and CysLTs, mediated by the augmented expression of the $CysLT_1$ receptor and subsequent $LTD_4$-triggered BSMC proliferation. IL-13 was also found to augment $CysLT_1$ expression in fibroblasts[59]. These findings support a role for CysLTs in the airway remodeling observed in asthmatic patients and may provide a rationale for preventive and therapeutic intervention.

We also found that B lymphocytes expressed $CysLT_1$ at both the mRNA and protein levels. Moreover, a two- to threefold enhancement of $CysLT_1$ expression was observed following B-cell exposure to a combination of activating anti-CD40 antibody and IL-4[60]. The expression of $CysLT_1$ was also enhanced when B lymphocytes were cocultured with CD154-transfected fibroblasts in the presence of IL-4. Moreover, IL-4 and CD40-activated B lymphocytes showed an increased responsiveness to $LTD_4$ in terms of cytosolic calcium flux, which was totally prevented by the selective $CysLT_1$ antagonist montelukast. Stimulation of IL-4 and CD40-activated B lymphocytes with picomolar concentrations of $LTD_4$ induced mature epsilon transcripts and up-regulated immunoglobulin (Ig)E and IgG production two- to threefold. Expression of $CysLT_1$ could thus be up-regulated in B lymphocytes following stimulation with CD154 and IL-4, with consequent increased responsiveness of the cells to $LTD_4$ in terms of Ig production. Early and colleagues[54] also recently reported that IL-4 could enhance $CysLT_1$ expression in B cells as well as T cells.

Dendritic cells (DCs) acquire, during their maturation, the expression of the chemokine receptor CCR7 and the ability to migrate to lymph nodes in response to CC chemokine ligand 19 (CCL19). This migration is impaired in mice lacking the $LTC_4$ transporter and restored by addition of exogenous $LTC_4$. To define the role of LT in human DC function, we studied the expression and function of the CysLT receptors during DC differentiation from monocytes and subsequent maturation[61]. Maturation of DC with LPS, a classic Toll-like receptor 4 agonist, reduced $CysLT_1$ expression by 50%, whereas $CysLT_2$ expression was increased. In contrast, the Toll-like receptor 3 agonist poly inosinic and cytidylic acid (polyI:C) had no effect on $CysLT_1$ or $CysLT_2$ receptor expression. Down-regulation of $CysLT_1$ expression by LPS could not be mimicked by TNFα alone or in combination with IL-1β or IL-6. It was, however, prevented by inhibitors of COX and could be reproduced by a combination of TNFα and prostaglandin $E_2$. Immature DCs and DCs matured with polyI:C, but not with LPS, responded to $LTD_4$ with a robust cytosolic calcium flux, which was prevented by the $CysLT_1$ antagonist montelukast. $LTD_4$ induced DC chemotaxis and enhanced DC migration in response to CCL19 in DCs matured with polyI:C, but only weakly in DCs matured with LPS. Our data suggest that human DCs may differentially respond to leukotriene, depending on their maturational stimuli.

Interestingly, $LTD_4$ priming of immature DC enhanced IL-4 production more than IFNγ production by naïve $CD4^+CD45RA^+$ T cells, with a twofold increase at 1 n*M* $LTD_4$. Similarly, $LTD_4$ priming of poly I:C-matured DC enhanced IL-4 production by naïve T cells fourfold. In contrast, LPS-matured DC were not affected by $LTD_4$ priming in inducing T-cell production of either IL-4 or IFNγ (unpublished observations).

### $CysLT_2$

$CysLT_2$ is coexpressed with $CysLT_1$ on most leukocytes, including neutrophils, eosinophils, basophils, mast cells, monocytes, macrophages, DCs, and B lymphocytes. It is also expressed in the absence of $CysLT_1$ in a number of cell types, including vascular smooth muscle cells and endothelial cells, heart Purkinje-conducting fiber cells, cardiomyocytes, and adrenal chromaffin cells[62].

$CysLT_2$ expression was shown to be modestly up-regulated by IL-4 in mast cells[31]. It was also up-regulated by IFNγ, but not by IL-1β, IL-4, IL-5, IL-13, or TNFα, in eosinophils from asthmatic patients[63]. However, we reported that $CysLT_2$ expression levels were not increased in human bronchial smooth muscle cells by IL-4, IL-13, TGFβ, or IFNγ [57]. Recently, Woszczek and colleagues[64] reported that IFNγ could up-regulate $CysLT_2$ expression in human endothelial cells with enhanced responsiveness of the cells to CysLTs. As with $CysLT_1$, $CysLT_2$ expression appears to be controlled differently in each cell type.

In conclusion, a number of cytokines can modulate the expression of $CysLT_1$ and/or $CysLT_2$ in different cell populations. This is in contrast to the receptors for $LTB_4$ and suggests that responsiveness of various cells to $LTC_4$ and $LTD_4$ may be quite dependent on environmental factors and subject to a finer degree of control.

Globally, as reviewed in this paper, LTs and cytokines are involved in a complex network of interactions, which appear to be ligand-, receptor-, and cell-specific. At this time, there are still very limited data available on the mechanisms and signaling pathways involved in the bidirectional modulation of cytokines and LTs. For instance, not all signaling by the G-protein coupled LT receptors is G-protein dependent. Moreover, several orthosteric receptor antagonists can act as inverse agonists and new allosteric ligands may provide precious tools to help understand LT receptor signaling. As we learn more about the interactions between LTs and cytokines, our understanding of this complex network should help us to devise more selective approaches for intervention in disease states where enhancement or reduction of lipid mediator and cytokine effects are sought.

## ACKNOWLEDGMENTS

The authors' work was supported by grants from the Medical Research Council of Canada, the Canadian Institutes of Health Research, the National Cancer Institute of Canada, the Society for Cancer Research, the Foundation for Research into Children's Diseases, and by scholarships and studentships from the Canadian Institutes of Health Research and the Fonds de la recherche en santé du Québec. MR-P is the holder of a Canada Research Chair in Inflammation.
